# Non-oncologic Total Femoral Arthroplasty: A Case Report

**DOI:** 10.7759/cureus.24487

**Published:** 2022-04-25

**Authors:** Max McCall, Andrew Corbett, Patricia Baumann

**Affiliations:** 1 Orthopedics, University of Central Florida College of Medicine, Orlando, USA; 2 Orthopedic Surgery, Largo Medical Center, Largo, USA; 3 Orthopedic Surgery, Bay Pines VA, Bay Pines, USA

**Keywords:** femoral shaft fractures, orthopedic procedure, atypical femur fracture, total joint arthroplasty, arthroplasty and trauma

## Abstract

As patient longevity continues to improve, the rate of lower limb revision arthroplasties will continue to increase as patients outlive the expiration of their implants. With continued bone loss and reduced stability, there is a limit to the number of revision operations that can be performed. Total femoral arthroplasty (TFA) is an increasingly popular limb-salvaging alternative that can restore some degree of daily function to patients. This report presents a 73-year-old male with multiple right lower-limb operations following two extreme motorcycle accidents in the last 22 years. Due to continued pain and poor femoral bone stock following multiple total knee arthroplasty (TKA) revisions, a TFA was performed. The procedure was successful and post-operative expectations were met despite setbacks in immediate rehabilitation. Overall, TFA is an effective alternative to lower limb amputation in the setting of aseptic, non-oncologic bone loss following multiple knee revisions. However, careful management is necessary to reduce the risk of infection and other complications.

## Introduction

Due to the result of an aging population, total knee arthroplasty (TKA) and total hip arthroplasty (THA) are being performed more frequently. As the longevity of patients increases, the need for knee or hip revisions increases due to a limited lifetime of the prosthetics implanted. If more revision operations are being performed within a patient’s lifetime, complications and bone loss are more likely to occur with each procedure. If too much bone loss occurs, the ability for a subsequent revision dwindles as the stability of the implant decreases. If a patient’s prosthetic is failing or causing significant discomfort but there is not enough bone stock for a revision, one common option is amputation.

However, total femoral arthroplasty (TFA) or total femoral replacement (TFR) are becoming a more popular alternative to salvage the lower limb [1]. This procedure involves complete replacement of the entire femur with a prosthetic implant. It was first described by Buchman in 1965 and has since been used for both oncologic and non-oncologic purposes [2]. Non-oncologic purposes include periprosthetic infection and aseptic bone loss due to frequent revisions or trauma [1]. Similar to in comparison to TKA or THA, a major benefit of TFA is the quick return in function compared to a lower-limb amputation [1].

Despite its growing popularity, TFA is still an incredibly arduous, technically challenging, and risky operation. These challenges lead to a high risk of complications including infection, loosening, and failure in both the short and long term. It is important to thoroughly assess the risks and benefits for each individual patient in the selection process. Post-operative expectations should be carefully explained prior to any decision to operate.

## Case presentation

We present a healthy 73-year-old male with an extensive medical history of the right leg. Back in 1999, the patient was in his first major motorcycle accident after colliding with another motor vehicle. The injuries from this accident eventually led to a fasciotomy, a popliteal-distal posterior tibial artery bypass, and an antibiotic-coated intermedullary nail in the tibia. Specifics about the fractures at the time are unknown. These treatments were initially successful until the patient presented with knee pain in 2009. A primary right TKA was performed at an outside facility due to arthritic changes at that time. This was effective in reducing his pain and enabling his return to functionality until a subsequent motorcycle accident in May 2018. There was significant damage to the distal femur with an open wound, so a temporary distal femoral replacement was performed. A more permanent secondary hinged TKA was then performed after risk of infection was reduced in June 2018 by an outside facility.

He was then seen in March 2019 by our clinic for continued right knee pain with evidence of aseptic loosening. A revision of the right hinged TKA was scheduled. However, the case was delayed until September 2019 due to a positive methicillin-susceptible Staphylococcus aureus (MSSA) swab and use of steroids prior to surgery. A revision of the femoral component of the hinged TKA was performed at that time. He was discharged on post-operative day (POD) 3 after walking 225 feet with the physical therapy team.

**Figure 1:**
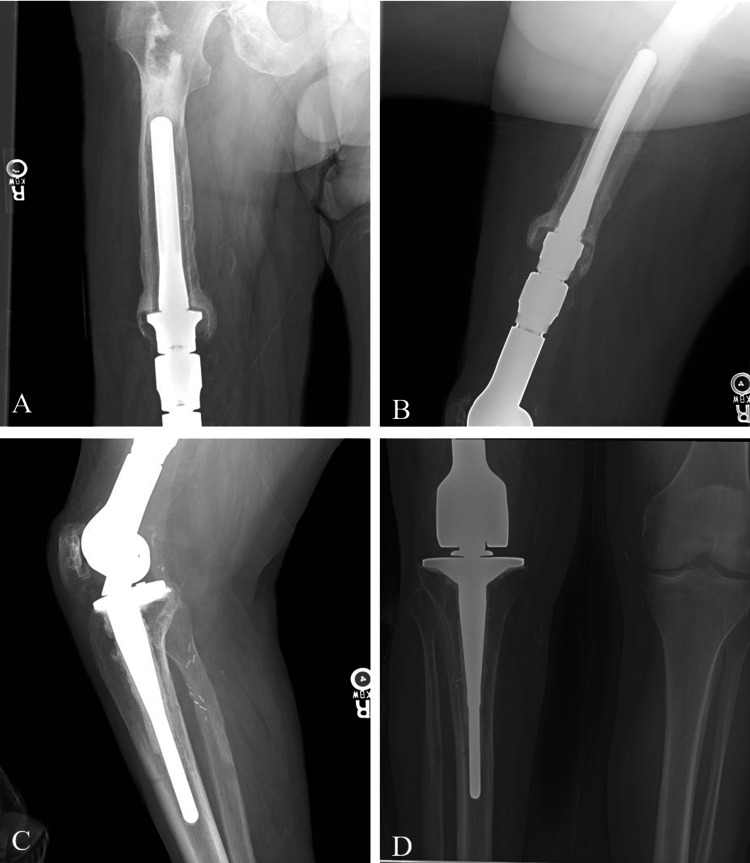
Pre-operative anterior-posterior (AP) x-rays of the thigh (A) and knee (D) as well as lateral x-rays of the thigh (B) and knee (C).

He then returned in October 2019 for his one-month follow up with continued leg pain despite non-steroidal anti-inflammatory drug (NSAID) use and his home exercise protocol. He was seen again in April, May, and July 2020 with continued thigh pain despite attempts at improvement with NSAIDs, numbing gel, physical therapy, and pulsed electromagnetic field (PEMF) therapy. Imaging at this time showed no visible changes with the prosthetics. The patient presented again in January and February 2021 in a wheelchair due to increased thigh pain. Evidence of aseptic loosening was noted in July when the patient was able to “shuck the components” while the knee was actively flexed causing the implant to disengage. A knee brace was provided and repeat x-ray imaging was performed. Imaging showed aseptic loosening of the right distal femoral replacement (Figure 1A-D).

At his following appointment in May 2021, a decreased range of motion (ROM) and a tender thigh was noted on physical exam. A conversion from a distal femoral replacement to a right TFA and right THA was scheduled as he had radiographic evidence of degenerative changes in the hip. Due to elective surgery restrictions as a result of the COVID-19 pandemic, the surgery was delayed again. His final pre-operative evaluation occurred in November 2021. At this point, he was cleared to undergo a conversion from a hinged right TKA revision to a right TFA and right THA scheduled for November 29, 2021.

Surgical procedure summary

Patient was given 1 gram of vancomycin, 2 grams of cefazolin, and tranexamic acid pre-operatively. After standard pre-operative preparation, an incision for a posterolateral approach of the hip was made. The soft tissue was dissected utilizing electrocautery and hemostasis obtained utilizing electrocautery. The tensor fascia lata was incised in line with the incision and retracted with Charnley retractor. A posterior capsular flap was performed. The hip was then dislocated. Utilizing an oscillating saw, the femoral cut was then performed. The acetabulum was properly exposed. Progressive reaming of the acetabulum was performed. The trial prosthetic was placed after anterior and posterior osteophytes were removed. The area was copiously irrigated with antibiotic laden pulsatile lavage and suctioned dry. The acetabular component was impacted in place and found to be well seated.

At this time, attention was turned to the femur. A trochanteric slide was performed for reattachment at the end of the procedure to aide with abduction. The femur was skeletonized for removal after the posterior approach to the hip was extended to a direct lateral approach of the femur. Once the femur was free, it was removed with the femoral hinge. The tibial component from his prior TKA was removed at this time.

Construction of a trial was performed on the back table with the removed tibial component. The trial was tested and demonstrated good knee and hip ROM. The trial was then removed so that the wound could be copiously irrigated with antibiotic lavage and chlorhexidine wash. The femoral component was then constructed with the proximal body of the total hip and the distal hinged knee. The femoral and tibial bushings were then attached to the femur with the axel and locking pin. This was reduced into the tibia. The femoral head was then impacted on the proximal body and the liner was impacted into the acetabulum. The hip was reduced with an audible click of the constrained head reducing into the constrained liner.

The knee and hip were taken through full ROM and found to be stable. The wound was again irrigated. The posterior capsular flap was reattached utilizing 2 fiber wire and the trochanteric slide was reattached to the femur with the fiber wire. This was done through the proximal body, around the slide, and into the posterior capsular flap. The gluteus maximus was reattached with 5 ethibond. The tensor fascia lata and soft tissue were approximated utilizing 2-0 undyed strata fix, while the skin was closed with a running 4-0 Monocryl.

Post-operative course

**Figure 2:**
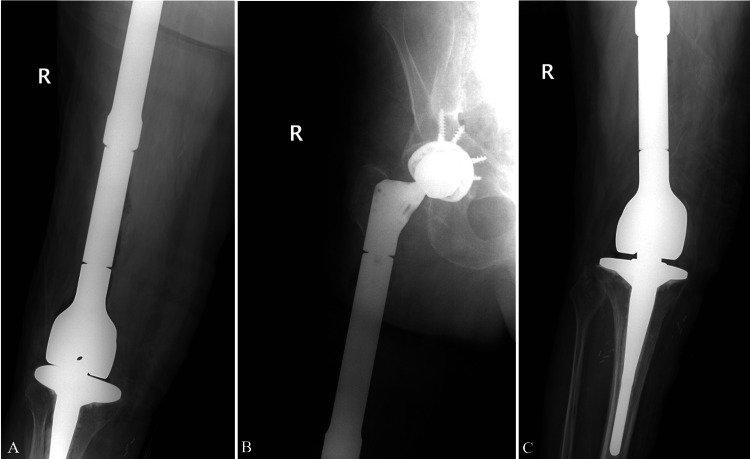
Immediate post-operative anterior-posterior (AP) x-rays of the femur (A), hip (B), and knee (C).

After the operation, flat plate anterior-posterior (AP) x-rays of the pelvis, femur, and knee were taken (Figure 2A-C). Another 2 grams of cefazolin was provided to the patient. His initial post-operative course was uneventful and was able to bear weight by POD 2. Pain was well-managed, and he was able to walk 15 feet with the physical therapy team. He was then transferred to the inpatient rehabilitation unit. Unfortunately, on POD 4, a rapid response was called due to hypovolemic shock during bowel movement. He was found to be in sinus tachycardia at 122 beats per minute with a blood pressure of 84/51 mmHg and a hemoglobin of 5.7 g/dL. No blood was seen in the stool at that time. Physical exam showed extensive ecchymoses at the right hip and scrotum. After receiving four blood transfusions over the next 24 hours in the medical ICU, the patient’s hemoglobin rose to 9.4 g/dL and blood pressure to 124/68 mmHg. His levels returned to baseline at that time, and he was admitted back to the rehabilitation floor on POD 7. The patient’s physical therapy was limited for the next several days due to the extensive swelling of his scrotum.

Eventually, he was able to work with physical therapy to begin walking the unit floor. On POD 17, he was discharged home and worked with home therapy. On POD 22, a sanguineous fluid discharge began from his incision. He was started on oral antibiotics and a negative pressure wound therapy system was applied. This has been effective without any evidence of infection locally or systemically. The patient was seen in the office on POD 29. He was ambulating 40 feet and is happy with his operation.

## Discussion

As the area of total joint arthroplasty continues to expand and evolve, there is a concomitant increase in the amount of revision arthroplasties as well. Initial projections predicted that total hip and knee revisions were projected to grow by 137% and 601% from 2005 to 2030, respectively [3]. More recent projections in some countries indicate a further increase of up to 90% in the incidence of revision arthroplasties over the next 30 years [4]. While it would be hopeful to expect that patients would require no more than one revisional operation, unexpected circumstances, such as those seen in this patient, can lead to multiple ones. Regardless of the reason for revision, the amount of bone stock available for implant stability is expected to decrease with each subsequent revision.

 In these patients, there are a reduced number of options. While TFA is still considered a radial limb salvage procedure compared to the alternative amputation, studies of non-oncologic TFA have shown a significant improvement in pain relief and functional ability. In one retrospective review of 59 patients undergoing TFA for end-stage prosthetic disease, 98% were able to walk again at almost 5 years [5]. In another study of 14 patients undergoing TFA, 50% of patients no longer required any analgesia post-operatively after all patients described moderate to severe pain prior [6]. In this same study, 93% described improvement in their mobility with Musculoskeletal Tumor Society (MSTS) scores improving from an average of 23% to 59%. A larger study that reviewed 100 non-oncologic TFAs showed an improvement in Eneking scores from 1.25 to 3.29 for the hip and 2.09 to 3.29 for the knee with an average of five years of follow up [7]. Another study showed improvements in Harris hip scores on average from 30.2 pre-operatively to 65.3 after two years [8].

 Despite these impressive improvements in pain and function, they need to be carefully considered against the risk of complications as seen in our patient’s case. In a study of 20 patients undergoing non-oncologic TFA, 25% experienced new infections and 30% required a revision after at least two years of follow up [8]. In another study of 100 TFA patients, deep infection was found in 12 while dislocation occurred in six [7]. The study on 14 patients undergoing TFA experienced three patients with infection while five experienced repeated dislocations [6]. These complications also need to be judged in the context of the risks for the alternative. Infections in post-lower limb amputation wounds have ranged from 13-40% [9] with a mortality rate in some studies proving 9% for below-knee amputations and 18% for above-knee amputations [10]. Regardless of the decision to proceed with either amputation or TFA, these studies heavily emphasize the need for proper patient selection and a strong multidisciplinary team both pre- and post-operatively.

## Conclusions

Primary THA and TKA, as well as revisions, require a certain amount of usable bone stock to provide long-term stability for the prosthetic implant. As patients undergo repeat arthroplasty procedures, develop bone cancer, or experience severe trauma, the amount of viable bone stock decreases considerably. Lower-limb amputations were historically the only option for these patients. However, TFA has become an effective third option for improvement in pain and function. Despite the apparent benefits of this limb-salvaging procedure, surgeons should still proceed with caution and consider its many risks. Our 73-year-old patient’s traumatic motorcycle history led to the need for a TFA as a radical limb-salvaging procedure. While his significant post-operative complications were concerning, their risk would also have been present if he had decided to proceed with an amputation. Overall, he is now progressing well with early weightbearing, improved mobility, and significantly reduced pain. 
